# Two new Neotropical species of *Ceracis* Mellié (Coleoptera, Ciidae) and redefinition of the *cucullatus* group

**DOI:** 10.3897/zookeys.66.1570

**Published:** 2011-10-03

**Authors:** Caio Antunes-Carvalho, Cristiano Lopes-Andrade

**Affiliations:** 1 Programa de Pós-Graduação em Entomologia, Departamento de Entomologia, Universidade Federal de Viçosa, 36570-000, Viçosa, Minas Gerais, Brazil; 2 Departamento de Biologia Animal, Universidade Federal de Viçosa, 36570-000, Viçosa, Minas Gerais, Brazil

**Keywords:** Ciid, minute tree-fungus beetle, Ciinae, Brazil, Mexico

## Abstract

Two new Neotropical species of *Ceracis* Mellié are described: *Ceracis cassumbensis* Antunes-Carvalho & Lopes-Andrade, **sp. n.** from a single locality in northeastern Brazil and *Ceracis navarretei* Antunes-Carvalho & Lopes-Andrade, **sp. n.** from a single locality in southern Mexico. Scanning Electron Microscope images of adults and photographs of holotypes and male terminalia are provided for both species, their similarities and differences with other *Ceracis* are briefly discussed, and the *cucullatus* species-group is redefined for including the new species described herein.

## Introduction

*Ceracis* Mellié (Coleoptera: Ciidae: Ciinae) encompasses 47 described species, being the second most speciose genus of the family. The genus was redefined by Lawrence (1967), who dealt mostly with Nearctic *Ceracis* but briefly discussed their affinities to Neotropical and Indo-Pacific species. He has also proposed two species-groups, the *furcifer* and the *cucullatus*, each including morphologically related species.

*Ceracis cucullatus* (Mellié), which names the *cucullatus* group, has drawn the attention of ciidologists due to its broad and disjunct geographic distribution. It is widespread in the Neotropical region, also occurring in several localities of the Afrotropical and Afrotemperate regions (sensu Morrone 2002), including several islands (Mellié 1849, Scott 1926, Blackwelder 1945, Lawrence 1967, Lopes-Andrade 2008, Lopes-Andrade et al. 2009, Lawrence and Lopes-Andrade 2010). There is a single record of the species from France (Abeille de Perrin 1874), but it is possibly not established there.

While conducting a survey on the morphology, life cycle and geographic distribution of *Ceracis cucullatus*, mainly to evaluate the conspecificity of disjunct populations under this name, we found two morphologically related new species. Here we describe *Ceracis cassumbensis* sp. n., a rare record of Ciidae in a Brazilian estuarine system, and *Ceracis navarretei* sp. n. from southern Mexico. We include them in the *cucullatus* species-group, which is redefined.

## Material and methods

Holotypes were neither dissected nor examined under Scanning Electron Microscope (SEM). SEM images of whole specimens (Figs 4-6, 14-16) and photographs of dissected sclerites of male terminalia (Figs 7-10, 17-20) are from topotypes (specimens collected in the type locality but not labeled as paratypes; sensu Evenhuis 2008). These figures are cited in the descriptions for the purpose of illustration.

Examination of specimens, measurements and descriptions were made under a Zeiss Stemi 2000 stereomicroscope with a scale ocular. Holotypes were photographed with a Canon EOS 1000D digital camera attached to the same stereomicroscope. Digital photographs taken from different focus were processed and enhanced in the image stacking freeware CombineZP (Hadley 2010). Permanent slide preparations of male terminalia followed the methodology detailed by Lopes-Andrade (2011) and were photographed with a Canon A640 digital camera adapted to a Zeiss Axioskop 40 compound microscope. SEM images were taken with a LEO 1430 VP. A few topotypes were dehydrated in a series of alcohol solutions, dried in a Critical Point Dryer (Balzers CPD 020), mounted on stubs and sputter-coated with gold (Balzers Sputter Module SCA 010).

The following abbreviations are used for measurements and ratios: CL, length of the antennal club; EL, elytral length (taken from the base of scutellum to the elytral apex); EW, greatest elytral width; FL, length of the antennal funicle; GD, greatest depth of the body (taken from the elytra to the metaventrite); PL, pronotal length along midline; PW, greatest pronotal width; TL, total length (EL+PL; head not included). Range, mean and standard deviation are given for the abovementioned measurements and the following ratios: EL/EW; EL/PL; GD/EW; PL/PW; TL/EW. The ratio GD/EW was adopted as an indication of degree of convexity, and TL/EW indicates degree of body elongation. These measurements and ratios were taken from the whole type series. Measurements of antennomeres, eyes, scutellum and abdominal ventrites were taken only from holotypes. Morphological variations between specimens of the type series (males and females) are given in the section on “Variation”, together with measurements and ratios (accompanied by mean + standard deviation). Specimens selected as holotypes are fully pigmented males.

We compared specimens of *Ceracis cassumbensis* sp. n.and *Ceracis navarretei* sp. n. withnamed specimens of *Ceracis cucullatus* from Brazil, Galapagos and several localities from Africa. Dissected terminalia of males from these localities were also carefully compared. The terminology adopted for external morphology and male terminalia's sclerites are explained by Lopes-Andrade and Lawrence (2005) and Lopes-Andrade (2008). The term sensillifer is used here to designate the compound sensory structure on the ciid antennal club (see Lawrence 1971, Lopes-Andrade and Lawrence 2005, Lawrence and Lopes-Andrade 2010). For a brief explanation on the use of the terms mesoventrite and metaventrite, see Lopes-Andrade (2007).

The following acronyms are used in this paper:

ANIC Australian National Insect Collection, CSIRO Ecosystem Sciences (Canberra, Australia)

CZUG	Colección Entomológica del Centro de Estudios en Zoología, Universidad de Guadalajara (Zapopan, Jalisco, Mexico)

LAPC	Cristiano Lopes-Andrade Private Collection (Viçosa, MG, Brazil)

## Descriptions

### Ceracis cassumbensis

Antunes-Carvalho & Lopes-Andrade sp. n.

urn:lsid:zoobank.org:act:26A7C976-D6E8-44C2-AC4A-A795421CAE24

http://species-id.net/wiki/Ceracis_cassumbensis

Figs 1 10 

#### Type-locality.

“Ilha da Cassumba” (Cassumba island) in Caravelas, southern portion of the state of Bahia, northeastern Brazil (17°46'S, 39°17'W).

#### Etymology.

The specific epithet refers to the *terra typica* of the species.

#### Diagnosis.

Each antenna with eight antennomeres. Pronotum with relatively fine punctation; its anterior edge projected for- and upward forming a raised plate, slightly concave, with a short emargination at apex. Elytral punctation relatively dense. First abdominal ventrite with a broad transversely oval, setose sex patch (Fig. 6, arrow). Tegmen with lateral edges bearing a small excavation near apex (Fig. 7, arrows).

#### Description.

**Figures 1–3.**
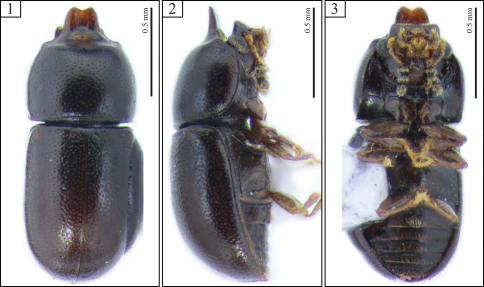
Habitus of *Ceracis cassumbensis* Antunes-Carvalho & Lopes-Andrade, sp. n., holotype. **1** Dorsal view **2** Lateral view **3** Ventral view.

**Figures 4–10.**
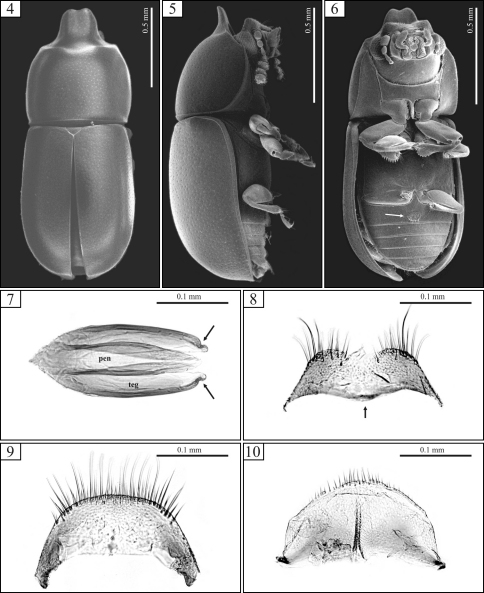
*Ceracis cassumbensis* Antunes-Carvalho & Lopes-Andrade, sp. n., SEM of male topotypes (4–6) and slide preparations of male terminalia of a topotype (7–10). **4** Dorsal view **5** Lateral view **6** Ventral view, showing the transversely oval sex patch at the first abdominal ventrite (arrow) **7** Aedeagus showing penis (pen) and tegmen (teg). Note the conspicuous excavation in either side of tegmen (arrows) **8** Eighth sternite with anterior margin rounded at middle (arrow) **9** Eighth tergite **10** Fused ninth and tenth tergites.

Male holotype (Figs 1-3), measurements in mm: TL 1.56; PL 0.60; PW 0.64; EL 0.96; EW 0.64; GD 0.56. Ratios: PL/PW 0.94; EL/EW 1.50; EL/PL 1.60; GD/EW 0.88; TL/EW 2.44. Body elongate, robust; dorsal and ventral surfaces dark brown, almost black; basal antennomeres and funicle, mouthparts and legs mostly yellowish brown; antennal club blackish and terminal palpomere of the maxillary palp yellowish black. Head barely visible from above; dorsal surface subglabrous, sparsely punctate, bearing a transverse impression at disc, preceded by a weak protuberance (seen in the dissected topotype); frontoclypeal ridge produced forward, transversely concave, with anterior margin emarginate at middle forming two subtriangular plates visible from below (Fig. 6), the anterior edge with a row of setae along it. Each eye with a widest diameter of 0.14 mm; some short slender yellowish setae emerging from the intersection between ommatidia. Each antenna with eight antennomeres (FL 0.09, CL 0.17, CL/FL 1.89); length of antennomeres (in mm) as follows (from base to apex): 0.07, 0.05, 0.05, 0.03, 0.02, 0.05, 0.05, 0.07; each antennomere of the club bearing several sparse slender setae, and four conspicuous sensillifers symmetrically positioned at its upper portion. Pronotum with sides reasonably rounded, widest at middle; lateral margins narrow, not visible from above, except for the most posterior corners; anterior edge projected for- and upward, forming a curved raised plate, slightly concave, with a short emargination at apex (Figs 1, 4); disc impressed in the area surrounding pronotal projection; anterolateral angles inconspicuously produced, relatively obtuse; punctation relatively fine, single, uniformly distributed, the posterior half of the median longitudinal surface devoid of punctures; distance between punctures from 1.75 to 2.25 puncture-widths, being greater at the anterior half of pronotum (including pronotal projection); each puncture bearing a fine yellowish decumbent minute seta; in between punctures shiny, microreticulate. Scutellum small, triangular, with few punctures, each one bearing a short, fine, decumbent bristle; basal width 0.11mm and length along the longitudinal midline 0.05 mm. Hind wings developed. Elytra with sides subparallel at the basal two-thirds, then abruptly converging toward apex; punctation single, confused, denser than pronotal punctation; punctures irregular, but ever finer than those on pronotum; vestiture similar to that of pronotum, but in between punctures smooth and shiny. Ventral sclerites microreticulate. Prosternum in front of coxae shallowly concave longitudinally, and a bit transversely convex; surface beside coxae weakly concave; prosternal process laminate, reasonably elevated, almost as long as coxae. Metaventrite moderately convex, bearing sparse slender setae; punctation shallow, consisting mostly of few punctures close to the lateral edges; median suture (discrimen) obscurely indicated posteriorly (see section on “variation”). Abdominal ventrites bearing sparse slender decumbent yellowish setae, longer than those on the dorsal surface; punctation shallow and sparse; lengths of abdominal ventrites (from base to apex, at the longitudinal midline) as follows (in mm): 0.19; 0.08; 0.08; 0.06; 0.06; length of abdominal ventrites together 0.46 mm; abdominal width (basal width of the first abdominal ventrite) 0.63 mm; first abdominal ventrite bearing a broadly transverse margined setose sex patch (Fig. 6, arrow), located postered of center, with a transverse diameter of 0.06 mm. Apex of each protibia expanded; outer apical angle rounded and bearing a row of spines.

**Male terminalia.** (Figs 7–10) Ninth segment (=genital ring) V-shaped. Fused ninth and tenth tergites (Fig. 10) with posterior margin rounded and bearing small suberect bristles at middle; sides slightly diverging, almost subparallel. Eighth sternite (Fig. 8) with posterior margin shallowly emarginate at middle; posterior angles rounded and bearing some bristles; lateral margins diverging; anterior margin biconcave, rounded and slightly sclerotized at middle but not forming a strut (Fig. 8, arrow). Eighth tergite (Fig. 9) with posterior margin almost straight, bearing long and short bristles along it; lateral margins diverging; anterior margin concave. Aedeagus (Fig. 7) around twice as long as wide; basal piece not observed, possibly membranous. Tegmen slightly longer than and twice as wide as penis; posterior portion subtriangular, then subparallel sided at most of its length, lateral edges slightly curved inward to apex; both sides bearing a small excavation near apex (Fig. 7, arrows). Penis elongate, subcylindrical; sides subparallel at the basal three-fourths, with apical one-fourth subtriangular and weakly sclerotized.

**Females.** Differing from males in the following features: frontoclypeal ridge rounded, not produced. Lateral margins of pronotum rounded; anterior margin rounded, not produced, bearing small yellowish setae along it; pronotal and elytral punctation slightly finer than in males. Abdominal sex patch absent.

#### Variation.

Males, measurements in mm (n=21, including holotype): TL 1.12–1.80 (1.46 + 0.18); PL 0.44–0.84 (0.66 + 0.11); PW 0.48–0.76 (0.63 + 0.07); EL 0.68–0.96 (0.80 + 0.07); EW 0.52–0.76 (0.64 + 0.07); GD 0.44–0.68 (0.55 + 0.06). Ratios: PL/PW 0.92–1.19 (1.04 + 0.07); EL/EW 1.12–1.33 (1.25 + 0.06); EL/PL 1–1.55 (1.23 + 0.14); GD/EW 0.76–0.92 (0.86 + 0.04); TL/EW 2.15–2.50 (2.28 + 0.09). Body varying from dark reddish brown to dark brown (almost black). Frontoclypeal ridge and apex of pronotum weakly developed in the smallest males and strongly projected in the largest ones. Discrimen indiscernible to barely discernible in most individuals.

Females, measurements in mm (n=10): TL 1.32–1.56 (1.45 + 0.09); PL 0.56–0.68 (0.62 + 0.05); PW 0.56–0.68 (0.61 + 0.05); EL 0.76–0.92 (0.84 + 0.05); EW 0.6–0.72 (0.66 + 0.04); GD 0.52–0.6 (0.56 + 0.04). Ratios: PL/PW 1–1.07 (1.01 + 0.02); EL/EW 1.17–1.44 (1.28 + 0.09); EL/PL 1.24–1.44 (1.36 + 0.08); GD/EW 0.81–0.94 (0.85 + 0.05); TL/EW 2.06–2.44 (2.22 + 0.12).

#### Type series.

Male holotype (LAPC) “BRASIL: BA Caravelas; Ilha da Cassumba 30.ii.2006 *leg.* K.S. Furieri, F.C.C. Barreto, E.S. Rediguieri” “*Ceracis cassumbensis* Antunes-Carvalho & Lopes-Andrade HOLOTYPUS” [printed on red paper]. Paratypes: 20 males, 10 females (LAPC), same data as holotype. All paratypes distinguished labeled “*Ceracis cassumbensis* Antunes-Carvalho & Lopes-Andrade PARATYPUS” [printed on yellow paper].

#### Natural history.

Cassumba is a continental island at the Caravelas-Peruípe estuarine system, with around 120Km^2^. It is located at the northern portion of the Atlantic Forest and encompasses forest remnants and large mangrove areas mixed in a landscape apparently well preserved. It is the first record of Ciidae from the island and a rare record of the family from a Brazilian estuarine system. However, we do not know either the host-fungus of this single collection of *Ceracis cassumbensis* sp. n. or whether it was caught close to a mangrove or a forest remnant at the island.

### Ceracis navarretei

Antunes-Carvalho & Lopes-Andrade sp. n.

urn:lsid:zoobank.org:act:63754F8E-972F-418F-988A-FA24504AFAA9

http://species-id.net/wiki/Ceracis_navarretei

Figs 11 20 

#### Type-locality.

Dos Amates, southern portion of the state of Veracruz, southern Mexico (17°24'N, 94°35'W).

#### Etymology.

The specific epithet is in honor of José Luis Navarrete Heredia, who made available to us the majority of the specimens included in the type series.

#### Diagnosis.

Body with very fine, sparse punctation. Each antenna with nine antennomeres. Pronotum mostly black; elytra and apex of pronotum reddish brown. Pronotal apex projected for- and upward, forming a curve, raised foursquare plate, weakly emarginated at the anterior edge. Elytra with lateral margins subparallel at the basal half, then gradually converging toward the apex. Aedeagus 4× longer than wide (Fig. 17); tegmen with parallel sides at most of their lengths, lateral edges angulate at the beginning of the apical third (Fig. 17, arrows) and then converging in straight line toward the apex.

#### Description.

**Figures 11–13.**
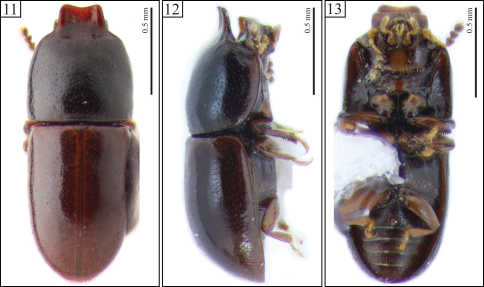
Habitus of *Ceracis navarretei* Antunes-Carvalho & Lopes-Andrade, sp. n., holotype. **11** Dorsal view **12** Lateral view **13** Ventral view.

**Figures 14–20.**
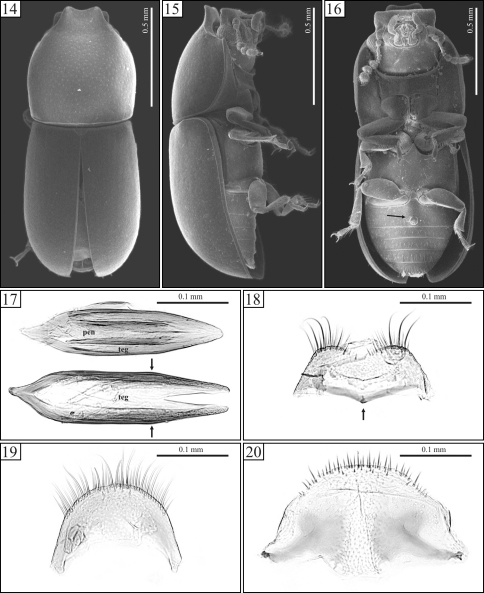
*Ceracis navarretei* Antunes-Carvalho & Lopes-Andrade, sp. n., SEM of male topotypes (14–16) and slide preparations of male terminalia of topotypes (17–20). **14** Dorsal view **15** Lateral view **16** Ventral view, showing the circular margined sex patch at the first abdominal ventrite (arrow) **17** Above, aedeagus showing penis (pen) and tegmen (teg). Below, a tegmen alone. Arrows indicate the angulation point from which the sides of tegmen converge in straight line toward the apex **18** Eighth sternite, showing the anterior margin weakly produced at middle (arrow) **19** Eighth tergite **20** Fused ninth and tenth tergites.

Male holotype (Figs 11-13), measurements in mm: TL 1.60; PL 0.72; PW 0.64; EL 0.88; EW 0.62; GD 0.56; TL/EW 2.58; PL/PW 1.13; EL/EW 1.42; EL/PL 1.22; GD/EW 0.90. Body subcylindrical, moderately convex; elytra and apex of pronotum reddish brown, remainder of pronotum black; ventral surface reddish brown; legs, mouthparts, basal antennomeres and funicle yellowish brown; antennal club dark brown. Head barely visible from above; dorsal surface flattened, subglabrous, bearing minute, sparsely decumbent fine setae, almost indiscernible; punctation sparse, consisting of shallow coarse punctures; frontoclypeal ridge produced forward, transversely concave, with its anterior margin slightly emarginate at middle, the anterior edge with a row of setae along it. Each eye with a widest diameter of 0.13 mm; some short slender yellowish setae emerging from the intersection between ommatidia. Each antenna with nine antennomeres (FL 0.09, CL 0.15, CL/FL 1.67); length of the antennomeres (in mm) as follows (from base to apex): 0.06, 0.04, 0.04, 0.02, 0.02, 0.02, 0.04, 0.04, 0.06; each antennomere of the club bearing several sparse slender setae, and four conspicuous sensillifers symmetrically positioned at its upper portion. Pronotum with subparallel sides, widest at middle; lateral margins narrow, being a bit thicker at the anterior portion; only the anterior and posterior corners can be seen from above, but the latter is weakly visible; anterior edge projected for- and upward, forming a curve, raised foursquare plate, slightly emarginated at apex (Figs 11, 14); raised plate transversely concave; anterolateral angles slightly produced, moderately obtuse; punctation fine, single, relatively uniform; distance between punctures from 2.5 to 3 puncture-widths; vestiture of yellowish decumbent seta; in between punctures shiny, microreticulate. Scutellum small, triangular, glabrous, with few fine punctures; basal width 0.11mm; length along the longitudinal midline 0.05 mm. Hind wings developed. Elytra with sides subparallel at basal half, then gradually converging to apex; only the most anterior corners visible from above; punctation single, confused, finer and denser than that of pronotum; vestiture consisting of minute, fine, decumbent yellowish setae; in between punctures smooth and shiny. Ventral sclerites microreticulate. Prosternum in front of coxae shallowly concave longitudinally and transversely convex; surface beside coxae weakly concave; prosternal process laminate, almost as long as coxae. Metaventrite moderately convex, bearing sparse slender setae; punctation shallow and sparse, almost indiscernible; discrimen indiscernible. Abdominal ventrites bearing sparse slender decumbent yellowish setae, longer than those on the dorsal surface; punctation shallow and sparse; lengths of abdominal ventrites (from base to apex, at the longitudinal midline) as follows (in mm): 0.19; 0.07; 0.07; 0.07; 0.08; abdominal length 0.50 mm, abdominal width (basal width of the first abdominal ventrite) 0.55 mm; first abdominal ventrite bearing a circular margined sex patch (Fig. 16, arrow), located postered of center, with a transverse diameter of 0.04 mm. Apex of each protibia expanded; outer apical angle rounded and bearing a row of spines.

**Male terminalia.** (Figs 17–20) Ninth segment (=genital ring) V-shaped. Fused ninth and tenth tergites (Fig. 20) with posterior margin reasonably straight, with small suberect bristles along it; sides diverging, each bearing a small protuberance at middle. Eighth sternite (Fig. 18) with posterior margin weakly emarginate at middle; posterior angles rounded and bearing some bristles; lateral margins diverging; anterior margin biconcave, sclerotized and forming a short median strut (Fig. 18, arrow). Eighth tergite (Fig. 19) with posterior margin rounded, bearing long and medium size bristles along it; lateral margins diverging; anterior margin concave. Aedeagus (Fig. 17) 4× longer than wide; basal piece not observed, possibly membranous. Tegmen slightly longer than and twice as wide as penis; posterior portion subtriangular and then parallel sided at most of its length, either side angulate at the beginning of the apical third (Fig. 17, arrows) and converging in straight line toward apex. Penis elongate, subcylindrical; sides subparallel at the basal three-fourths, with apical one-fourth subtriangular and weakly sclerotized.

**Females.** Differing from males in the following features: frontoclypeal ridge rounded, not produced. Head with dorsal surface usually convex. Lateral margins of pronotum rounded; anterior margin rounded, not produced; pronotal and elytral punctation slightly finer than in males. Abdominal sex patch absent.

#### Variation.

Males, measurements in mm (n=22, including holotype): TL 1.22–1.74 (1.53 + 0.13); PL 0.46–0.78 (0.67 + 0.08); PW 0.50–0.68 (0.61 + 0.05); EL 0.76–0.96 (0.86 + 0.05); EW 0.50–0.68 (0.60 + 0.05); GD 0.40–0.60 (0.53 + 0.04). Ratios: PL/PW 0.92–1.27 (1.09 + 0.08); EL/EW 1.38–1.57 (1.44 + 0.05); EL/PL 1.14–1.65 (1.31 + 0.12); GD/EW 0.80–0.94 (0.88 + 0.04); TL/EW 2.43–2.73 (2.56 + 0.09). Color of pronotum varying from black to reddish brown, usually reddish; elytra dark reddish to reddish brown. Anterior edge of pronotum weakly developed in the smallest males and strongly projected in the largest ones. In some cases the anterior and posterior corners of the lateral margins of pronotum are not visible from above. Surface of pronotum weakly to distinctly microreticulate. Eighth sternite with anterior margin completely rounded to weakly produced at middle.

Females, measurements in mm (n=18): TL 1.16–1.50 (1.35 + 0.09); PL 0.44–0.58 (0.51 + 0.04); PW 0.44–0.60 (0.54 + 0.04); EL 0.72–0.92 (0.84 + 0.05); EW 0.46–0.62 (0.55 + 0.04); GD 0.42 + 0.54 (0.49 + 0.04). Ratios: PL/PW 0.88–1.00 (0.95 + 0.04); EL/EW 1.43–1.59 (1.51 + 0.05); EL/PL 1.54–1.79 (1.64 + 0.07); GD/EW 0.81–0.96 (0.89 + 0.05); TL/EW 2.31–2.57 (2.43 + 0.08).

#### Type series.

Male holotype (CZUG) “MEXICO: Veracruz Dos Amates 03.vi.1988 S.L. Álavez *leg*.” “*Ceracis navarretei* Antunes-Carvalho & Lopes-Andrade HOLOTYPUS” [printed on red paper]. Paratypes: 19 males, 16 females (11 males and 12 females at CZUG, 8 males and 4 females at LAPC), same data as holotype; 2 females and 2 males (2 females and 1 male at ANIC, 1 male at LAPC) “MEXICO: Veracruz Dos Amates 28/2/1987 polypore 0114 J. Navarrete”. All paratypes distinguished labeled “*Ceracis navarretei* Antunes-Carvalho & Lopes-Andrade PARATYPUS” [printed on yellow paper].

#### Natural history.

Dos Amates is surrounded by small villages, being a mosaic of forest remnants and deforested areas apparently far from major urban areas. We have no information on the host-fungus of this new species. We only know that a few specimens were collected in a polypore (see “Type series” above).

## Discussion

Organizing morphologically similar species of Ciidae into species-groups has been an useful taxonomic tool, especially in speciose genera as *Ceracis*, *Cis* Latreille and *Scolytocis* Blair (Lopes-Andrade 2008, Lopes-Andrade et al. 2002), as it facilitates the task of recognizing new species or synonyms. Currently, there are four defined species-groups (*cucullatus*, *furcatus*, *furcifer* and *singularis*) for 17 species of *Ceracis*. The *furcatus* group includes *Ceracis furcatus* (Bosc), *Ceracis militaris* Mellié, *Ceracis minutus* Dury and *Ceracis variabilis* (Mellié). These species were discussed together in the work of Lawrence (1967) and called a species-group by Lopes-Andrade (2002), who erroneously included *Ceracis furcifer* Mellié (lapsus calami with *Ceracis minutus*; Lopes-Andrade pers. obs.). *Ceracis furcifer* names another group including *Ceracis cornifer* (Mellié), *Ceracis cylindricus* (Brèthes), *Ceracis furcifer*, *Ceracis hastifer* (Mellié), *Ceracis monocerus* Lawrence, *Ceracis ruficornis* Pic, *Ceracis simplicicornis* (Pic) and *Ceracis unicornis* Gorham (sensu Lawrence 1967). The *singularis* group (sensu Lopes-Andrade et al. 2002) includes *Ceracis furcicollis* (Blair), *Ceracis limai* Lopes-Andrade et al. and *Ceracis singularis* (Dury).

Lawrence (1967) proposed the *cucullatus* group for *Ceracis bicornis* (Mellié) and *Ceracis cucullatus*. He also synonymized *Ceracis bilamellatus* (Pic), *Ceracis lamellatus* (Pic) and *Ceracis tabellifer* (Mellié) with *Ceracis cucullatus* because they were described based on size and development of pronotal characters in the male, features considered variable within the populations examined by the author. *Ceracis cucullatus* and *Ceracis bicornis* can be distinguished by the pronotal apex that is weakly emarginate in *Ceracis cucullatus*, and deeply emarginate in *Ceracis bicornis* forming two distinct horns in males with developed secondary sexual characteristics. Lawrence (1967) suggested that the nearctic *Ceracis thoracornis* (Ziegler) and the palearctic *Ceracis shikokuensis* (Miyatake) and *Ceracis japonus* (Reitter) could be part of the *cucullatus* group, although not formally including them. We have examined named specimens of these three species and concluded that they are not similar enough to either *Ceracis cucullatus* or *Ceracis bicornis* to be included in the group. Here we redefine the *cucullatus* group so to include *Ceracis cassumbensis* sp. n. and *Ceracis navarretei* sp. n., as follows: (i) each antenna with eight or nine antennomeres, (ii) pronotum with fine and sparse punctation, (iii) body moderately long, and (iv) relatively long lamina on the apex of pronotum in males with fully developed secondary sexual characteristics. In the original proposal (see Lawrence 1967), only species with nine antennomeres were included in the *cucullatus* group. However, the number of antennomeres can vary even among morphologically similar species of *Ceracis*, as within the *furcifer* group: *Ceracis furcifer* and *Ceracis ruficornis* have eight antennomeres, while the other species have nine antennomeres (Lawrence 1967).

Among the species in the *cucullatus* group, as proposed here, *Ceracis navarretei* sp. n. is possibly the most similar to *Ceracis cucullatus*, mainly to its African populations. Differences are notable especially on male terminalia: The tegmen of *Ceracis navarretei* sp. n. has the lateral edges parallel at most of their lengths, the apical third converging in straight line toward the apex.In named specimens of *Ceracis cucullatus* examined by us, the sides of tegmen are either subparallel or weakly curved. Moreover, the aedeagus in *Ceracis navarretei* sp. n.is 4× longer than wide, while in *Ceracis cucullatus* it is around 3×. *Ceracis cassumbensis* sp. n. may be distinguished from *Ceracis cucullatus* by its greater depth of the body (most evident when comparing females), antennae with eight antennomeres, elytral punctation denser and abdominal sex patch larger and transversely oval (Fig. 6, arrow). Moreover, either lateral edge of the tegmen in *Ceracis cassumbensis* sp. n. has a peculiar excavation near apex (Fig. 7, arrows). This characteristic is also observed in *Ceracis similis* Horn, although this species is distinguishable from *Ceracis cassumbensis* sp. n. by its reddish body, punctation comparatively coarser and denser and relatively wider pronotal lamina.

The morphological limits of both *Ceracis cucullatus* and *Ceracis bicornis* were not satisfactorily established. The former is one of the most widely distributed ciid species in the tropics and the latter is widespread in the Neotropical region, having been reported in Mexico, Guatemala, Costa Rica, Peru (Lawrence 1967), northeastern, southeastern and southern Brazil (C. Lopes-Andrade pers. obs.). Morphological variation among allopatric populations of these species has been frequently observed and possibly interpreted as polymorphism, which may be overshadowing the recognition of new species. The description of *Ceracis cassumbensis* sp. n. and *Ceracis navarretei* sp. n. is a reflex of this scenario. *Ceracis cucullatus* and *Ceracis bicornis* may be cryptic species complexes and shall be more carefully studied. Other *Ceracis* species, as those in the *furcatus* group, also have strong morphological interpopulational variation and possibly involve undescribed forms.

## Supplementary Material

XML Treatment for Ceracis cassumbensis

XML Treatment for Ceracis navarretei
